# Pregnancy outcomes after vaginal probiotic supplementation before frozen embryo transfer: a randomized controlled study

**DOI:** 10.1038/s41598-023-39078-6

**Published:** 2023-07-23

**Authors:** Isarin Thanaboonyawat, Sootthinan Pothisan, Somsin Petyim, Pitak Laokirkkiat

**Affiliations:** Infertility Unit, Department of Obstetrics and Gynecology, Faculty of Medicine, Siriraj Hospital, Mahidol University, 2 Wanglang Road, Siriraj, Bangkoknoi, Bangkok, 10700 Thailand

**Keywords:** Randomized controlled trials, Clinical microbiology, Clinical pharmacology, Infertility

## Abstract

In women receiving assisted reproductive treatment, intrauterine lactobacilli dominance has been associated with higher rates of pregnancy achievement. This randomized controlled trial conducted in the fertility clinic of the university hospital from 7 August 2019 to May 2021, aimed to compare the clinical outcome of embryo transfer in frozen-thaw cycles with Lactobacillus supplementation prior to embryo transfer and the standard treatment. A total of 340 infertile women underwent randomization. The biochemical and clinical pregnancy rates were comparable between the groups (39.9 and 34.2% in the study group vs. 41.8 and 31.7% in the control group); however, the miscarriage rate was significantly decreased in the study group (9.5 vs. 19.1%, respectively, *p* = 0.02), [OR = 0.44, 95% CI (0.23, 0.86)]. Among 49 women diagnosed with bacterial vaginosis, the live birth rate in the study group was higher than the control group (42.31 vs. 26.09%, *p* = 0.23), [OR = 2.08, 95% CI (0.62, 6.99)]. In the blastocyst transfer group (n = 206), the live birth rate was significantly higher in the study group than in the control group (35.71 vs. 22.22%, *p* = 0.03) [OR = 1.9, 95% CI (1.05, 3.59)]. Therefore, intravaginal lactobacilli supplementation before embryo transfer in the frozen-thaw cycle did not improve the biochemical and clinical pregnancy rate in the general population but significantly reduced the miscarriage rate.

Trial Registration: TCTR20190429001 (29/04/2019) @ www.thaiclinicaltrials.org.

## Introduction

Probiotics may be beneficial in multiple situations, including assisted reproduction technology. *Lactobacillus*, a probiotic and normal flora, resides in the vagina and endometrial cavity. It produces hydrogen peroxide^1,2^, bacteriocin^3^, and lactic acid to maintain vaginal pH of less than 4.5^4^. This environment inhibits the growth of pathogenic organisms. Cihat Ünlü and colleagues found that *Lactobacillus* supplementation can inhibit various pathological organisms, except *Candida albicans,* by producing organic acids and hydrogen peroxide^4^. Lactobacilli may be beneficial for implantation due to their ability to inhibit pathogen growth by producing organic acids and hydrogen peroxide, controlling pH, and adhering to epithelial cells^5^.

In women receiving assisted reproductive treatment, intrauterine lactobacilli dominance has been associated with higher rates of pregnancy achievement^6^. A recent study used endometrial fluid or vaginal aspirate and 16S rRNA gene pyrosequencing-based technique^7^ or next-generation sequencing to explore the endometrial microbiome^8^. The microbiota profile has been categorized into Lactobacillus-dominated and non-Lactobacillus dominated with significant adverse reproductive outcomes observed in non-Lactobacillus-dominated women^7,9,10^. A recent retrospective study investigated 2285 women undergoing their first fresh autologous IVF cycles. A lower live birth rate and higher preterm birth rate were found in the non-Lactobacillus-dominant group^11^. Nevertheless, Hashimoto reported a comparable pregnancy rate between the Lactobacillus dominated (> 80%) and dysbiotic endometrium^12^. Moreover, *L. miners* has been associated with normal early pregnancy, while *Lactobacillus crispatus* was found to be abundant in early miscarriage cases^13^. The dominance of certain bacterial strains may be associated with the pregnancy rate^1^. A 2021 study by Fernandez et al. reported an overall successful pregnancy rate of 56% in women with reproductive failure following 6 months of oral *Ligilactobacillus salivarius* CECT5713 treatment^14^. However, the absence of a control group makes this result less meaningful. The effects of probiotic supplementation on vaginal or endometrial colonization and the inhibition of pathogens^15^ is a relatively new area of research. Therefore, whether lactobacillus supplementation may restore the normal flora, increase implantation and pregnancy rates, and reduce the miscarriage rate, merits further investigation^12^.

The *Lactobacillus* proportion was reportedly low after menstruation, however, could slowly increase throughout follicular development and reached its highest during the luteal phase^16^. However, studies of endometrial bacteria indicate that the diversity, quantity and ratios of microorganisms may not be regulated by hormones between the days of LH + 2 and LH + 7^7^. Some studies suggested that the vaginal microbiome can change within some days or months^17^. This alteration may be affected by various factors, including vaginal microbiome supplements. Moreover, the suggested duration of treatment for bacterial vaginosis (BV) using lactobacilli is six days^4,18^. Therefore, six days of probiotic supplementation may be the appropriate duration to change the vaginal microbiota and allow some lactobacilli to migrate into the endometrial cavity. In an earlier trial, women receiving IVF used only three days of vaginal probiotics and no benefit was observed^1^. This result may be explained as follows. In the fresh cycle, ovarian stimulation and progesterone supplementation can reduce the proportion of *Lactobacillus* while increasing *Prevotella* and *Atopobium* colonization in the endometrial cavity^8^. However, during their study procedures, a normal saline vaginal douche may have interfered with the results by removing both the normal flora and pathogens, reversing the environment, and improving the pregnancy rate^1^. Consequently, the study of frozen-thaw cycles may be helpful to reduce the effects of ovarian stimulation and vaginal douching.

We designed this study to compare the biochemical pregnancy rate between women using intravaginal probiotic supplementation and those with standard treatment before embryo transfer (ET) in the frozen-thaw cycles. The primary outcome of the study was to compare the biochemical pregnancy rate between the study and control groups. The secondary outcomes were to compare the implantation rate, miscarriage rate, clinical and ongoing pregnancy rate, and live birth rate. *Lactobacilli acidophilus* was used in the study since it can produce hydrogen peroxide and organic acid to regulate the vaginal PH and can adhere to the epithelial cells. All these characteristics are possibly beneficial for implantation.

## Materials and methods

### Study population

This randomized controlled study was approved by the Institutional Review Board, Faculty of Medicine Siriraj Hospital (SIRB) and all research was performed in accordance with relevant guidelines/regulations. The study was conducted at the Siriraj Hospital infertility unit from August 2019 to March 2021. It was registered at https://www.thaiclinicaltrials.org (TCTR20190429001-29/04/2019). Infertile Thai women aged 18–39 years who were attending the frozen-thaw cycles were enrolled. Women who had a failure of implantation after three consecutive transfers of good quality embryos, endometrial thickness < 7 mm, or were allergic to the medicine (such as a burning sensation after using) used in the study group, Gynoflor (Medinova AG, Zurich, Switzerland), were excluded. Each participant was enrolled in the research once only. Gynoflor contains lyophilised, viable *Lactobacillus acidophilus* KS400 bacteria [100 million colony-forming units (cfu) per tablet] and 0.03 mg estriol. Each woman gave informed consent before participating in this study. Embryo transfer (ET) was not undertaken if no survived embryo was available, the patient was exposed or developed Covid-19, was waiting for surgery due to identifying the large endometrial polyp, receiving antibiotics, or was lost to follow-up.

### Endometrial preparation and embryo transfer

The endometrium was prepared with 4 mg daily oral estradiol hemihydrate (Estrofem, Novo Nordisk A/S, Denmark) which commenced on Day 2 of the menstrual cycle. The dosage was adjusted according to the endometrial thickness^19^. Transvaginal ultrasound (Aloka ProSound Alpha 7; Hitachi Healthcare Americas, Inc., Twinsburg, Ohio, USA) was performed to access endometrial thickness. Endometrial thickness ≥ 7 mm with a triple-layer appearance was acceptable for ET. All eligible patients were randomized by computerized blocks of four randomizations on the day that endometrial thickness attained the desired characteristics. The allocation ratio was 1:1. The allocation codes were held in sealed envelopes that were opened by a research nurse. The attending physicians, embryologists and statisticians were blinded to the randomization. The study group received one tablet of intravaginal lactobacilli (Gynoflor, Medinova AG, Zurich, Switzerland) daily for six days, starting the same day of luteal phase support. All patients received intravaginal micronized progesterone (Cyclogest®; Actavis, Barnstaple, UK) 800 mg per day for 3- or 5-days of progesterone priming before ET. On the day of ET, patients were asked to hold back their urine and lay in the lithotomy position. ET was performed under transabdominal ultrasound guidance. The sagittal view of the uterus was identified after speculum insertion. One or two, day 3 or day 5 embryos were transferred using a soft catheter (K-JETS-7019-SIVF; Cook, Bloomington, Ind., USA). The embryos were placed 1–2 cm away from the uterine fundus. Only good quality embryos, namely cleavage embryos with ≥ 6 blastomeres and < 10% fragmentation and blastocyst grade 4BB or higher, were transferred. The luteal phase support with micronized progesterone was provided for all participants until the pregnancy was confirmed. The pregnant women received luteal phase support until reaching the 10th week of gestation.

### Vaginal discharge collection

On the last day of the endometrial thickness evaluation, a resident physician collected vaginal discharge before transvaginal sonography was performed. The color, pH, and odor of vaginal discharge were recorded. A wet smear was performed. All information was interpreted after the pregnancy result was revealed. The presence of budding yeast and/or pseudohyphae was accepted as candidiasis. Amsel’s criteria were used to diagnose bacterial vaginosis. All investigation results were blinded to the attending physicians. BV is more prevalent in tubal than non-tubal infertility. Because of the heterogenicity of BV prevalence reported in the infertile population and the non-association with decreased conception rate^20^, BV is not routinely screened in patients prepared for embryo transfer; therefore, no patients were treated specifically.

### Embryo warming and culture procedures

#### Day 3 embryos

The thaw media was pre-warmed in the incubator at 37 °C. The vitrified embryos were removed from the liquid nitrogen and the outer straw was removed immediately. The containing media in the inner straw was gently poured into thaw media 1 which contained 1 M sucrose in PBS. After 60 s in thaw media 1, the embryos were transferred to thaw media 2 which contained 0.5 M sucrose in PBS. Three minutes later, the embryos were put in thaw media 3 containing PBS only for another five minutes^21^. Then, the Day 3 embryos were cultured in Sydney IVF blastocyst medium (K-SIBM-50, Cook Medical, Inc., Bloomington, IN, USA) at 37 °C under 5% O_2_, and 6% CO_2_ for about 2 h before the transfer.

#### Blastocysts

The embryos were placed in 37 °C thaw media 1 (0.5 M sucrose in DPBS) for three minutes. Then, they were transferred to thaw media 2 (0.25 M sucrose in DPBS) and thaw media 3 (0.125 M sucrose in DPBS) for three minutes each in that order^22^. Finally, the blastocysts were cultured in the Sydney IVF blastocyst medium (K-SIBM-50, Cook Medical, Inc., Bloomington, IN, USA) at 37 °C under 5% O_2_ and 6% CO_2_ for about two hours before the transfer.

### Participants follow up after pregnancy confirmed

Serum beta hCG was evaluated 14 days after embryo transfer. Biochemical pregnancy was defined as the elevation of serum hCG (> 25 µg/dL). If pregnancy was confirmed, transvaginal sonography was performed to identify a gestational sac. Transvaginal sonography was then performed every two weeks until the 12th week of gestation. All patients received routine antenatal care until delivery. Clinical pregnancy was the visualization of an intrauterine gestational sac by transvaginal ultrasound at 3–4 weeks post-ET. The implantation rate was the number of gestational sacs observed divided by the number of embryos transferred^23^. Patients with an alive intrauterine fetus at GA 12 weeks were categorized as an ongoing pregnancy. Ectopic pregnancy was a pregnancy outside the uterine cavity, diagnosed by transvaginal sonography or surgical visualization. Miscarriage was defined as the spontaneous loss of either biochemical or clinical pregnancy that occurs before 20 completed weeks of gestation. Live birth was defined as live delivery post-24 weeks gestation.

The pregnancy outcomes were defined as followings:

Biochemical pregnancy rate = the number of patients with hCG > 25 IU/mL/the number of patients who received embryo transfer.

Clinical pregnancy rate = the number of patients with visible gestational sac/the number of patients who received embryo transfer.

Ongoing pregnancy rate = the number of patients with viable pregnancy at 12 weeks gestation/the number of patients who received embryo transfer.

Miscarriage rate = the number of patients with miscarriage/ the number of patients who received embryo transfer.

Live birth rate = the number of patients with live birth / the number of patients who received embryo transfer.

### Statistical analysis

There’s no previous study using lactobacilli supplementation during frozen-thaw ET cycles. Therefore, we hypothesized that the pregnancy rate in the intervention group would increase to 50% from the baseline (control group) rate of 34.2% in our clinic. To detect this difference with a significance level of 0.05 and a power of 0.8, a sample size of 153 patients per group is needed. Considering a 10% loss to follow-up for each additional cycle, 170 patients were included in each group. Statistical analyses were performed by using IBM SPSS Statistics for Windows (version 21.0; IBM Corp., Armonk, N.Y., USA). All tests of significance were two-tailed with a *p* value < 0.05. The demographic and baseline characteristics of the two groups including demographic data, reproductive history, and underlying disease of the patients were analyzed using the Student’s *t*-test, the chi-squared test and odds ratio as appropriate. Continuous data with normal distribution were presented as mean ± SD; otherwise, median and interquartile ranges were used.

### Ethics approval

The study was approved by the Institutional Review Board of a Siriraj hospital, Mahidol University.

## Results

**Figure 1:**
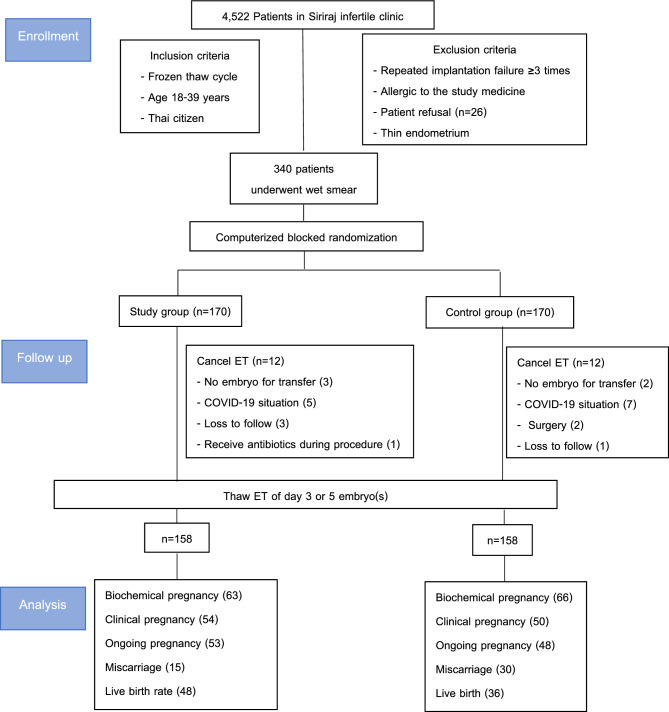
Flowchart of the study.

A total of 340 women in the infertility clinic underwent randomization and 24 did not receive ET, twelve in each group. In the study group, three patients had no remaining embryos, five patients were unable to attend treatment because of the COVID-19 situation, one received antibiotics for pharyngitis, and three patients did not come to the clinic for follow-up. In the control group, two patients had no survival embryos for transfer, seven patients were unable to attend treatment because of the COVID-19 situation, two were awaiting surgery, and one was lost to follow-up. Thus, the analysis was performed on 316 patients (158 in the study group and 158 in the control group) (Fig. 1).

The baseline characteristics, including the patients’ age, history of gravida/parity/miscarriage, duration of infertility, the number of previously conducted ET cycles, duration of endometrial preparation, level of beta-hCG, the embryo age, and the number of embryos transferred, were similar in both groups (Table1). The most common underlying diseases, namely endometriosis, myoma uteri, PCOS, endometrial polyp, and history of ectopic pregnancy of women, were similar in both groups, as well. The prevalence of endometriosis was 19.3%; the most common female infertility factor in this study. Male factors, such as azoospermia, oligospermia, asthenospermia et al., were found in 11.7%. The infertility causes were displayed in Table 2.

**Table 1:** Baseline characteristics of the population.

Characteristics	Study group (n = 158)	Control group (n = 158)	*p* Value
*Patients*			
Age (year)	35.10 ± 3.38	35.51 ± 3.25	0.28
Gravida (%)			0.29
0	62.03	67.09	
1	28.48	22.15	
2	8.23	6.96	
≥ 3	1.27	3.80	
Parity (%)			1
0	87.34	87.34	
≥ 1	12.66	12.66	
Previous Abortion (%)			0.65
< 3 times	98.73	98.10	
≥ 3 times	1.27	1.80	
Infertile period (months)	29.00 (18.00, 57.50)	32.5 (20.00, 54.25)	0.53
Previous ET times			0.25
0	87	86	
1	51	49	
2	20	19	
3	0	4	
Endometrial preparation (day)	17.35 ± 3.58	17.42 ± 3.22	0.86
Endometrial thickness (mm)	9.56 ± 1.39	9.56 ± 1.44	0.98
Embryonic stage (%)			0.24
Cleavage stage	37.97	31.65	
Blastocyst stage	62.03	68.35	
Number of embryos	1.68 ± 0.47	1.66 ± 0.48	0.63
Bacterial Vaginosis (%)	16.46	14.56	0.64
Fungus (%)	7.59	5.70	0.50

*ET* embryo transfer.

Data are mean ± SD, n (%),median (quatile range), or unless otherwise specified.

**Table 2:** Infertility causes.

	Study group (n = 158)		Controlled group (n = 158)		*p* Value
Factor	n	%	n	%	
*Female factor*					
Pelvic endometriosis	35	22.2	33	20.9	0.94
Tubal and pelvic	32	22.3	34	21.5	
Endometriotic cyst	23	14.6	22	13.9	
Anovulation	2	1.3	4	2.5	
Uterine	2	1.3	1	0.6	
Donor egg	2	1.3	1	0.6	
Cervical stenosis	0	0.0	1	0.6	
Male factor	44	27.8	51	32.3	

All the infertility causes were comparable in both groups.

Women who received lactobacilli had biochemical and clinical pregnancy rates of 39.9 and 34.2%, compared with 41.8 and 31.7% in the control group (*p* = 0.73, 0.63, respectively). The BV prevalence was 17.7% (30/170) and 14.1% (24/170), in the study and control groups, respectively. There were no statistically significant differences between the two groups in the implantation rate, ongoing pregnancy rate, ectopic pregnancy rate, multiple pregnancy rate, and live birth rate (Table 3). However, the miscarriage rate was found to be significantly lower in the study group (9.5 vs. 19.1%, *p* = 0.02), (OR = 0.44, 95% CI [0.23, 0.86]). The biochemical pregnancy rate was significantly higher in women that received Day 5 embryo transfer than in Day 3 embryo transfer (45.2 vs. 32.7%, *p* = 0.03). Nevertheless, the clinical pregnancy and live birth rates were similar (Table 3).

**Table 3:** Clinical outcomes.

Outcomes	Study group (n = 158)	Control group (n = 158)	*p* Value	Odds ratio (95% CI)
	N (%)	N (%)		
Primary outcome				
Biochemical pregnancy	63 (39.9)	66 (41.8)	0.73	0.92 (0.59, 1.45)
Clinical pregnancy	54 (34.2)	50 (31.7)	0.63	1.12 (0.70, 1.79)
Secondary outcomes				
Implantation rate	24.8	21.4	0.35	1.21 (0.81, 1.82)
Ongoing pregnancy rate	53 (33.5)	48 (30.4)	0.55	1.16 (0.72, 1.86)
Miscarriage rate	15 (9.5)	30 (19.1)	0.02†	0.44 (0.23, 0.86)
Ectopic pregnancy	1 (0.7)	1 (0.6)	0.95	–
Multiple pregnancies	12 (7.6)	6 (3.8)	0.15	2.08 (0.76, 5.69)
Live birth rate	48 (30.4)	36 (22.8)	0.13	1.48 (0.89, 2.45)

The chemical pregnancy rate, clinical pregnancy rate, implantation rate, ongoing pregnancy rate, ectopic pregnancy rate, multiple pregnancies, and live birth rate were comparable between the groups. However, the miscarriage rate of the total study population was significantly lowered in the study group.

**Figure 2:**
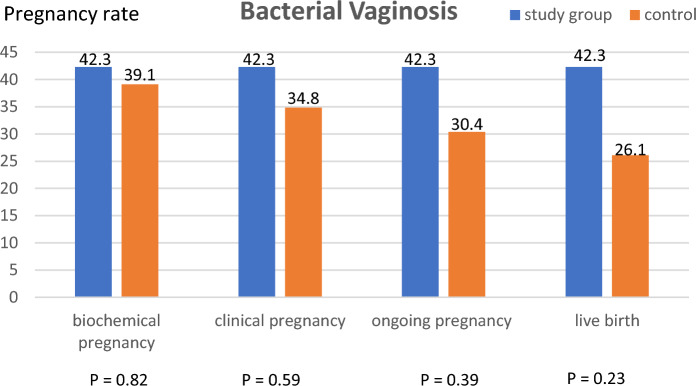
Pregnancy rate and live birth rate in the subgroup analysis of patients with bacterial vaginosis (n = 46) were presented. The biochemical, clinical, and ongoing pregnancy rates were comparable. The live birth rate was 1.5-times that in the study group, however, was not statistically significant.

**Figure 3:**
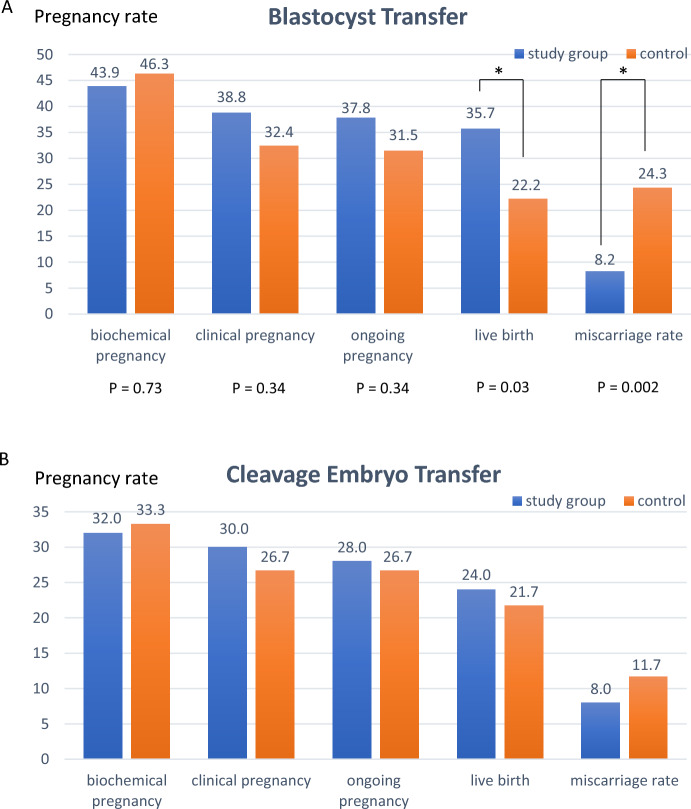
(**A**) Pregnancy rate and live birth rate in the subgroup analysis of patients who received blastocyst transfer. The biochemical, clinical, and ongoing pregnancy rates were comparable. However, the live birth rate was higher in the study group (35.7 vs. 22.2%, *p* = 0.03) [OR = 1.9, 95% CI (1.05, 3.59)], and the miscarriage rate was lower in the study group (8.2 vs. 24.3%, *p* = 0.002) [OR = 0.28, 95% CI (0.12, 0.65)]. (**B**) The subgroup analysis in cleavage embryo group showed that all the pregnancy outcomes were comparable in both groups (*p* > 0.05).

In a post-hoc subgroup analysis of 49 women diagnosed with BV, the clinical pregnancy rate in the study group (n = 26) was higher than in the control group (n = 23) (42.3 vs. 34.8%, *p* = 0.59). Moreover, the live birth rate in the study group was more than 1.5-times that in the control group (42.3 vs. 26.1%, *p* = 0.23) [OR = 2.08, 95% CI (0.62, 6.99)] (Fig. 2). However, both were not statistically significant. In women with vaginal candidiasis, the clinical pregnancy rate and live birth rate were comparable in both groups. In the subgroup of blastocyst transfer, the miscarriage rate was reduced (8.2 vs. 24.3%, *p* = 0.002) [OR = 0.28, 95% CI (0.12, 0.65)], and the live birth rate was significantly higher in the study group than in the control group (35.7 vs. 22.2%, *p* = 0.03) [OR = 1.9, 95% CI (1.05, 3.59)] (Fig. 3A). However, in the cleavage embryo group, all the outcomes were comparable (Fig. 3B).

## Discussion

A lactobacilli-predominant vaginal and endometrial environment has been associated with a higher pregnancy rate^24,25^ and a lower miscarriage rate^13^. We studied the clinical outcomes of intravaginal probiotic supplementation before ET during the frozen-thaw cycle. The biochemical and clinical pregnancy rates were comparable in both groups; a result that is consistent with a report from Gilboa and colleagues that showed a similar pregnancy rate in fresh ET cycles^1^. In contrast, other studies have reported that unfavorable endometrial receptivity due to a non-*Lactobacillus* dominated microbiome significantly decreases the implantation rate, pregnancy rate, ongoing pregnancy rate, and live birth rate^7,11,24,26^.

One meta-analysis suggested that probiotics could reduce the amount of abnormal microbiota (Gardnerella and Atopobium) and increase the quantity of the *Lactobacillus* genus^27^. The different clinical outcomes may be caused by the use of different types of probiotics, varying dosages, and the duration of supplementation^28^. Yet, there is no consensus on the appropriate dosage and duration of lactobacilli supplementation for changing the proportions of endometrial *Lactobacillus*. It has been suggested that four weeks of intravaginal probiotic supplementation should be able to change the endometrial microbiome from non-*Lactobacillus* dominant to *Lactobacillus*-dominant group^29^. Nevertheless, we used six days of lactobacilli supplementation as suggested by Moreno and colleagues^7^, understanding that intravaginal *Lactobacillus* dominance might not reflect the condition of the endometrial microbiome^7,16^. More studies are needed to investigate the bacterial shift in the uterine cavity after vaginal or oral lactobacilli supplementation.

We conducted a post hoc subgroup analysis to further explore pregnancy outcomes. Among the women who were diagnosed with BV, the clinical pregnancy rate, and the live birth rate were higher but did not reach statistical significance. Nevertheless, the increased live birth rate was clinically significant. The *Lactobacillus* supplementation may have helped reduce the low implantation and pregnancy rates caused by BV^16,29^. However, the small sample size in the subgroup analysis reduced our power to detect small differences. Some evidence suggests that the increase of pathological intravaginal organisms is associated with dysbiosis and leads to infertility^30,31^. Egbase et Al. showed that women who had a pathological organism in the endometrium had a lower implantation rate compared with women who had received antimicrobial agents and those without pathological organisms (18.7 vs. 41.3% and 38.1%; *p* < 0.01, respectively)^32^. Moreover, patients with chronic endometritis, which is associated with low implantation rate and unreceptive endometrium^33^, were found to contain fewer *Lactobacilli* and more abundant *Dialister, Bifidobacterium, Prevotella, Gardnerella*, and *Anaerococcus*^34^*. Lactobacilli* can balance the intravaginal ecosystem and inhibit pathological organisms^4,27,35^, such as *Gardnerella vaginalis* and *Escherichia coli,* which may be associated with failure of implantation^1,6^. However, we were not able to assess the endometrial microbiome in our study.

Subgroup analysis in the blastocyst group found that the live birth rate was higher in the study group (*p* = 0.03), despite similar biochemical and clinical pregnancy rates. The miscarriage rate was 24.3 and 8.2% in the control and study group, respectively (*p* = 0.002). Moreover, the increased live birth rate and decreased miscarriage rate were clinically significant. On the contrary, the clinical outcomes were comparable in the cleavage embryo group.

We transferred the blastocyst on day 6 of lactobacilli supplementation, while another study transferred fresh embryos on day 3^1^. Longer periods of lactobacilli supplementation before embryo transfer may more effectively shift the microbiome of the endometrial cavity to become *Lactobacillus* dominant. This may be beneficial for embryo survival, implantation and prevention of miscarriage. A 2021 study by Yoshida et al. suggested that *Lactobacillus* may promote trophoblast invasion, therefore increasing the implantation rate^36^.

Despite the comparable pregnancy rate, we observed a statistically significant reduction in the miscarriage rate in the study group. This was consistent with Moreno et al., who reported a decreased miscarriage rate from 60 to 16.7% in the probiotic supplement group^9,30^. An imbalance of intravaginal organisms predominated by a reduction of lactobacilli and an increase of other organisms, especially *Gardnerella vaginalis,* and some anaerobic bacteria, might explain the loss of some pregnancies^4,37^. In a recent study that hypothesized the microbiota composition in patients with live birth is the physiological scenario, *Lactobacillus* was found to be lower than the physiologic level in patients who were not pregnant and who experienced clinical miscarriage^38^. A deficit of hydrogen peroxide-producing *Lactobacillus* has been reported to reduce the implantation rate and increase the miscarriage rate in the first six weeks of pregnancy^39^. A study of bacteria in the transfer catheter tip also found that an increased number of *Lactobacillus* was associated with a significantly higher live birth rate (50%) compared with no *Lactobacillus* (21%)^24^. In a study of 35 women who underwent IVF, the predominant *Lactobacillus* group (> 90%) had higher implantation, pregnancy, clinical pregnancy, and birth rates (60.7, 70.6, 58.8, and 58.8%) than the control group (23.1, 33.3, 13.3, and 6.7%)^30^. Therefore, *Lactobacillus* may play a role in embryo transplantation^24,30^.

No patients in our study group had an adverse event related to the lactobacilli supplementation. This is in line with an earlier report of 50 pregnant women diagnosed with BV and treated with *Lactobacillus* plus low-dose estriol for six days where no negative effects on the pregnancy or the fetus were observed^4^. Although estriol is one of the components of the medicine used in the study group, estriol cannot transform into other forms of estrogen. It has a weak affinity to combine with estrogen receptors. Thus, it has a negligible effect on the endometrium under estradiol priming^40^.

This study is the first single-center, randomized controlled trial comparing the pregnancy outcome of ET in the frozen-thaw cycles between the Lactobacilli supplementation and a control group. No placebo group was one of the limitations of this study. Nevertheless, the study group demonstrated good compliance with the protocol. A wet smear was performed to diagnose BV in all participants, enabling the estimation of BV prevalence in infertile Thai women. Lactobacilli supplementation, therefore, may be beneficial for this specific group. However, we were not able to evaluate the health of the vaginal or endometrial microbiome, which has been associated with endometrial receptivity and the pregnancy rate^12,41^. Studies of the effect of lactobacilli supplementation on the endometrial microbiome are needed. In this study, both day 3 and day 5 embryos were included. The implantation and pregnancy rates of different embryo stages have differed. A benefit of probiotics supplementation was seen in blastocyst transfer. Therefore, future investigation in the blastocyst transfer group is preferred over day 3 embryo transfer. We included patients aged 18–39 years old, whose pregnancy rates were less influenced by oocyte quality compared with those aged > 39. Therefore, caution should be made using these results in advanced-aged patients.

## Conclusion

We found no effect of intravaginal *Lactobacilli* supplementation in the general IVF population, on the biochemical, clinical, ongoing pregnancy rate, implantation rate, and live birth rate. However, the intravaginal probiotic supplementation significantly reduced the miscarriage rate and increased the live birth rate in patients who received blastocyst transfer. Further research on the dose, duration and effect of *Lactobacillus* supplementation and an improved understanding of the endometrial microbiome before and after ET are needed.

## Data Availability

The study protocol and the anonymized data that underlie the results reported in this article are available to be shared. Researchers who request access must provide a methodologically sound proposal that has been approved by an independent review committee. A data-sharing statement between Siriraj Hospital, Mahidol University, and the requesting researcher’s institute will be required before data can be provided. The corresponding author, Isarin Thanaboonyawat will help to process data sharing.
